# Supplementary material for: Methanogenesis associated with altered microbial production of short-chain fatty acids and human-host metabolizable energy
Source: ISME J. 2025 May 22;19(1):wraf103. doi: 10.1093/ismejo/wraf103 (PMC12156016; doi:10.1093/ismejo/wraf103)
Supplement: Blake_NIH_Project_-_Methanogen_Paper_-_Supplememtary_v6_wraf103 [file blake_nih_project_-_methanogen_paper_-_supplememtary_v6_wraf103.docx]

**Below is supplementary material for the manuscript “Methanogenesis associated with altered microbial production of short-chain fatty acids and human-host metabolizable energy”**

Blake Dirks^1,2^, Taylor L. Davis^1,2^, Elvis A. Carnero^3^, Karen D. Corbin^3^, Steven R. Smith^3^, Bruce E. Rittmann^2,4^ & Rosa Krajmalnik-Brown^1,4*^

^1^Biodesign Center for Health through Microbiomes, Arizona State University, Tempe, AZ, USA. ^2^Biodesign Swette Center for Environmental Biotechnology, Arizona State University, Tempe, AZ, USA. ^3^AdventHealth Translational Research Institute, Orlando, FL, USA. ^4^School of Sustainable Engineering and the Built Environment, Arizona State University, Tempe, AZ, USA.

* Corresponding author: dr.rosy@asu.edu

**Supplementary Table 1: qPCR primer and themocycler details**

| **Group** | **Gene** | **Primers** | **Thermocycler Settings** | **Reference** | **Standards from** |
| --- | --- | --- | --- | --- | --- |
| Homoacetogens | *acsB* | ACS_f  ACS_r | 95°C 10 s, 52°C 20 s, 72°C 30 s, 40 cycles | [1] | *Blautia hydrogenotrophica*  DSM 10507 |
| Sulfate-reducing bacteria | *dsrA* | DSR1-F DSR-R | 95°C 10 s, 60°C 60 s, 35 cycles | [2] | *Desulfovibrio piger*  isolate FI11049 |
| Methanogens | *mcrA* | mlas  mcrA-rev | 95°C 30 s, 55°C 45 s, 72°C 30 s, 40 cycles | [3] | *Methanobrevibacter smithii*  ATCC 35061 |


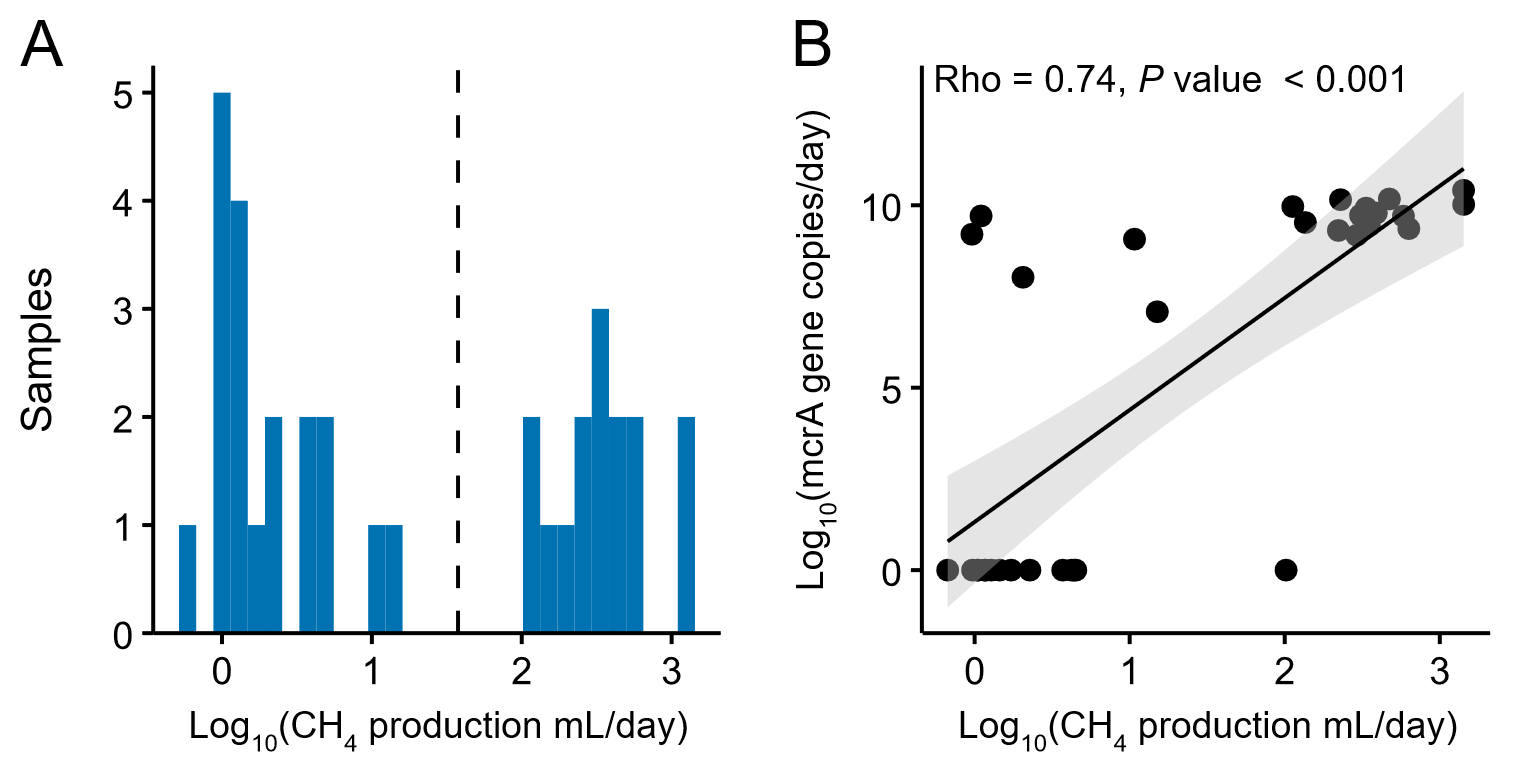


**Supplementary Figure 1. Distribution of CH_4_ production and the correlation between CH_4_** **production and daily fecal *mcrA* gene copy numbers.** A) The distribution of the log-transformed CH_4_-production rate. The dotted line marks the threshold for grouping participants as high or low CH_4_ producers at 1.57 (37.6 mL/day). B) The correlation between CH_4_-production rate and fecal *mcrA* gene copy numbers; the correlation had a *P* value < 0.001, indicating a strong and significant correlation between the two variables.

**Supplementary Table 2. Linear Mixed Model Variables**

| \| Independent Variables (IV): \| \| \| --- \| --- \| \| ·         CH_4_ Producer Group (high and low) \| \| \|  \|  \| \| Outcome Variables (OV): \| \| \| ·         Total fecal SCFAs normalized to PEG recovery (μg/day) \| \| \| ·         Fecal acetate normalized to PEG recovery (μg/day) \| \| \| ·         Fecal propionate normalized to PEG recovery (μg/day) \| \| \| ·         Fecal butyrate normalized to PEG recovery (μg/day) \| \| \| ·         Total Serum SCFAs (ng/mL) \| \| \| ·         Serum Acetate (ng/mL) \| \| \| ·         Serum Propionate (ng/mL) \| \| \| ·         Serum Butyrate (ng/mL) \| \| \| ·         Host Metabolizable Energy (%) \| \| \| ·         Gene copy number of key hydrogenotroph genes \| \| \| ·         Gene transcript abundance of key hydrogenotroph genes \| \| \|  \|  \| \| Covariates: \| \| \| ·         Randomized diet assignment \| \| \| ·         Diet Period \| \| \| ·         Diet Sequence \| \| \| ·         Colonic Transit Time (CTT) \| \| \|  \|  \| \| Random Factor: \| \| \| ·         Participant ID (Accounts for non-independence of the samples) \| \| |
| --- | --- | --- | --- | --- | --- | --- | --- | --- | --- | --- | --- | --- | --- | --- | --- | --- | --- | --- | --- | --- | --- | --- | --- | --- | --- | --- | --- | --- | --- | --- | --- | --- | --- | --- | --- | --- | --- | --- | --- | --- | --- | --- | --- | --- | --- | --- | --- | --- |

**Calculations for daily average H_2_ consumption for methanogenesis and the succinate pathway**

We calculated the average amount of H_2_ consumed by methanogenesis and propionate production in participants with detectable methanogen fecal copy numbers. We assumed that all propionate production went through the H_2_-consuming succinate pathway and that 95% of microbially produced propionate was absorbed in the colon, with 5% of microbially produced propionate remaining in the feces [4].

| **category** | **constant** | **value** | **units** |
| --- | --- | --- | --- |
| standard conditions | pressure | 1 | atm |
|  | universal gas constant | 0.08206 | (L atm)/(K mol) |
|  | temperature | 298 | K |
| Stoichiometry (equations below) | H_2_/CH_4_ stoichiometry coefficient | 4 | mol H_2_/ mol CH_4_ |
|  | H_2_/C_3_H_6_O_2_ stoichiometry coefficient | 2 | mol H_2_/ mol C_3_H_6_O_2_ |
| molecular weight | propionate molecular weight | 74.079 | g C_3_H_6_O_2_ / mol C_3_H_6_O_2_ |
| assumption 2 | fraction of produced propionate in feces | 0.05 | g C_3_H_6_O_2_ feces / g C_3_H_6_O_2_ total |

Methanogenesis stoichiometry

$$4H_{2}+CO_{2}\to CH_{4}+2H_{2}O$$

Succinate-pathway propionate production stoichiometry

$$C_{3}H_{4}O_{3}+2H_{2} \to C_{3}H_{6}O_{2}+H_{2}O$$

| **category** | **calculated variable** | **value** | **unit** |
| --- | --- | --- | --- |
| methane | daily avg methane production volume | 365.13 | mL CH_4_ / d |
|  | daily avg methane production mols | 0.01 | mol CH_4_/ d |
|  | daily avg hydrogen consumption mols | 0.06 | mol H_2_ / d |
| fecal propionate | daily avg fecal propionate mass | 121.33 | mg C_3_H_6_O_2_ feces / d |
|  | daily avg propionate production mass | 2.43 | g C_3_H_6_O_2_ / d |
|  | daily avg propionate production mols | 179.76 | mol CH_6_O_2_ / d |
|  | daily avg hydrogen consumption mols | 359.51 | mol H_2_ / d |

References

1. Gagen EJ, Denman SE, Padmanabha J et al*.* Functional gene analysis suggests different acetogen populations in the bovine rumen and tammar wallaby forestomach. *Appl Environ Microbiol* 2010;**76**:7785–95. 10.1128/AEM.01679-10

2. Leloup J, Loy A, Knab NJ et al. Diversity and abundance of sulfate-reducing microorganisms in the sulfate and methane zones of a marine sediment, Black Sea. *Environ Microbiol* 2007;**9**:131–42. 10.1111/j.1462-2920.2006.01122.x

3. Steinberg LM, Regan JM. Phylogenetic comparison of the methanogenic communities from an acidic, oligotrophic fen and an anaerobic digester treating municipal wastewater sludge. *Appl Environ Microbiol* 2008;**74**:6663–71. 10.1128/AEM.00553-08

4. Marcus A, Davis TL, Rittmann BE et al*.* Developing a model for estimating the activity of colonic microbes after intestinal surgeries. *PLoS One* 2021;**16**. 10.1371/journal.pone.0253542
